# Mitochondria as secretory organelles and therapeutic cargos

**DOI:** 10.1038/s12276-023-01141-7

**Published:** 2024-01-04

**Authors:** Joonho Suh, Yun-Sil Lee

**Affiliations:** Department of Molecular Genetics, School of Dentistry and Dental Research Institute, Seoul National University, Seoul, Republic of Korea

**Keywords:** Mitochondria, Drug development

## Abstract

Mitochondria have been primarily considered intracellular organelles that are responsible for generating energy for cell survival. However, accumulating evidence suggests that mitochondria are secreted into the extracellular space under physiological and pathological conditions, and these secreted mitochondria play diverse roles by regulating metabolism, the immune response, or the differentiation/maturation in target cells. Furthermore, increasing amount of research shows the therapeutic effects of local or systemic administration of mitochondria in various disease models. These findings have led to growing interest in exploring mitochondria as potential therapeutic agents. Here, we discuss the emerging roles of mitochondria as extracellularly secreted organelles to shed light on their functions beyond energy production. Additionally, we provide information on therapeutic outcomes of mitochondrial transplantation in animal models of diseases and an update on ongoing clinical trials, underscoring the potential of using mitochondria as a novel therapeutic intervention.

## Mitochondrial transplants: a novel approach to disease treatment

Mitochondria, known as the powerhouses of cells, are crucial for maintaining cellular balance. However, the methods by which cells release mitochondria into the extracellular space and how recipient cells utilize these mitochondria are still not fully understood. This study, by Joonho Suh and Yun-Sil Lee at Seoul National University in South Korea, explores the process of mitochondrial secretion and its potential therapeutic applications. The researchers reviewed the current understanding of mitochondrial secretion, focusing on the release of mitochondria as an entire organelle. They discovered that cells can secrete mitochondria via various methods, and these extracellular mitochondria can be absorbed by recipient cells to boost their functions. The study also emphasized the potential of using mitochondrial transplantation as a therapeutic strategy in various diseases. The authors concluded that understanding the methods of mitochondrial secretion and transfer could lead to new therapeutic strategies.

This summary was initially drafted using artificial intelligence, then revised and fact-checked by the author.

## Introduction

Mitochondria are multifaceted organelles that perform various functions to regulate cellular homeostasis^1^. Despite being the most well-known site of energy production or the “powerhouse” of the cell, mitochondria play many other pivotal roles, such as controlling the biosynthesis of molecules needed for cell growth and regulation of apoptosis^2^, intracellular calcium level^3^, redox balance^4^, the immune response^5^, cell stemness^6^, and interorganelle communication^7^. Mitochondria are also highly dynamic organelles, continuously changing their shapes through fusion and fission events^8,9^. The coordinated remodeling of mitochondrial morphology is tightly coupled with the major mitochondrial functions listed above, and imbalances in mitochondrial dynamics lead to mitochondrial dysfunction and pathological conditions^9^. A notable feature of mitochondria is their ability to generate mitochondrial-derived vesicles (MDVs) that transport mitochondrial components to lysosomes^10^, endosomes^11^, or peroxisomes^12^ for communication^13^. Emerging evidence suggests that MDVs and mitochondria may also be involved in intercellular communication or systemic regulation of cellular function^14–16^, the mechanisms of which are currently under active investigation.

Cells release diverse membrane-bound vesicles into the extracellular space to eliminate or transfer specific compounds or communicate with other cells^17^. Depending on their size and biogenesis pathway, these extracellular vesicles (EVs) are subcategorized as exosomes (50–150 nm in diameter, multivesicular body-derived), ectosomes (less than 0.1 μm to several μm in diameter, plasma membrane-derived), microvesicles (0.1-1 μm in diameter), large oncosomes (>1 μm in diameter), apoptotic bodies (>1 μm in diameter, apoptotic cell-derived), migrasomes (0.5–3 μm in diameter, migrating cell-derived), and the newly identified exomers (<50 nm in diameter, biogenesis unclear)^17–20^. However, simple categorization based on size and biogenesis pathway does not fully reflect the heterogeneity of the cellular origins, cargos, and functions of EVs. In this regard, the relatively recent discovery of extracellular mitochondria and EVs containing mitochondrial components that are secreted by many cell types adds a new level of complexity to EV biology^21^. The characterization, sorting and secretory mechanisms, and biological effects of extracellular mitochondria and EVs containing mitochondrial components under physiological or pathological conditions are currently under intense research.

Accumulating evidence suggests that extracellularly secreted mitochondria are transferred to recipient cells to induce therapeutic responses, suggesting that exogenous supplementation with mitochondria isolated from proper donor cells or tissues could be a therapeutic strategy. Local or systemic delivery of isolated mitochondria or mitochondrial transplantation has shown promising outcomes in animal models under various conditions, and several clinical trials involving mitochondrial transplantation to treat myocardial ischemia, cerebral ischemia, or inflammatory muscle diseases have been initiated. Despite significant attention and efforts aimed at developing mitochondrial transfer/transplantation strategies, research on the mechanisms of mitochondrial transfer is still in its early stages, and many critical questions remain unanswered. Importantly, understanding the mechanisms and biological effects of the extracellular secretion and transfer of mitochondria in vivo, as well as the mechanisms of recipient cell contact and uptake of extracellular mitochondria, will aid in the selection of appropriate sources for mitochondrial isolation and improve target specificity for successful mitochondrial transplantation therapy with minimal adverse effects.

In the first part of the review, we will discuss the evidence, mechanism and outcomes of extracellular mitochondrial secretion, focusing on the release of whole mitochondria due to its relevance with mitochondrial transplantation therapy involving the isolation and administration of intact mitochondria. We will not discuss the release of selective mitochondrial components (mitochondrial proteins, lipids, RNAs and/or DNA) or other modes of intercellular mitochondrial transfer such as tunnelling nanotubes (TNTs) as EVs containing mitochondrial components and TNT-mediated mitochondrial transfer have been previously reviewed^21–23^. In the second part, we will review the therapeutic effects of mitochondrial transfer in animal models under pathological conditions and current updates on human trials involving mitochondrial transplantation.

## Extracellular mitochondrial secretion and its biological effects

### Evidence and mechanisms of extracellular mitochondrial secretion

Mitochondria have been reported to be secreted extracellularly by many cell types, including mesenchymal stem cells (MSCs), astrocytes, neural stem cells, platelets, adipocytes, hepatocytes, cardiomyocytes, endothelial progenitor cells, osteoblasts, and various cell lines (Table 1). In this section, we briefly discuss key evidence and major mechanisms of mitochondrial extrusion.

**Table 1:** Evidence of extracellular mitochondrial secretion.

Cell type (species)	Conditions	Secreted form	Mechanism	Biological effect	Methods to visualize mitochondrial transfer	Reference
MSCs (human)	Standard culture conditions	Vesicles containing mitochondria	NR	Further investigation needed Possibly assists aerobic respiration of recipient cells	Donor cell mitochondria labeled with the DsRed2-mito transgene Recipient cells unlabeled	Spees et al., 2006^24^
MSCs (human)	Standard culture conditions Coculture with macrophages	Microvesicles or multivesicular bodies containing mitochondria	Undergo mitophagy and use ARMMs to release mitochondria	Manage intracellular oxidative stress by unloading depolarized mitochondria Enter macrophages to enhance mitochondrial bioenergetics	Donor cell mitochondria labeled with MitoTracker Red Recipient cell mitochondria labeled with MitoTracker Green	Phinney et al., 2015^25^
MSCs (human)	Standard culture conditions	Vesicles containing mitochondria	NR	Enter monocyte-derived macrophages and enhance macrophage oxidative phosphorylation to promote phagocytosis and suppress proinflammatory cytokine secretion	Donor cell mitochondria labeled with MitoTracker Red Recipient cell mitochondria labeled with MitoTracker Green	Morrison et al., 2017^40^
MSCs (rat)	Standard culture conditions	Vesicles containing mitochondria, MFN2, and PGC-1α	Secretion increased after PGC-1α overexpression	Enter intestinal epithelial cells and promote mitochondrial fusion and biogenesis, thereby improving mitochondrial metabolism and intestinal barrier function	Donor cells transfected with the RFP-mito plasmid Recipient cell mitochondria labeled with MitoTracker Red	Zheng et al., 2021^35^
AdMSCs (mouse)	Standard culture conditions	Exosomes containing mitochondria and mtDNA	NR	Enter alveolar macrophages and improve mitochondrial function Shift macrophages to anti-inflammatory phenotype	Donor cell mitochondria labeled with MitoTracker Red Recipient cell mitochondria labeled with the HSP60 antibody	Xia et al., 2022^41^
BMSCs (human)	Coculture with macrophages using transwell system	Vesicles containing mitochondria	NR	Enter macrophages and enhance phagocytosis	Donor cell mitochondria labeled with MitoTracker Red Recipient cells stained for CD45	Jackson et al., 2016^66^
BMSCs (human)	Coculture with macrophages	Vesicles containing mitochondria	nSMase pathway	Enter macrophages and enhance phagocytosis	Donor cell mitochondria labeled with MitoTracker Green Recipient cells unlabeled	Ko et al., 2020^67^
BMSCs (human, mouse)	Standard culture conditions Stimulation with mitochondrial stress-inducing or protective agents	Vesicles containing mitochondria	Further investigation needed Agents that affect mitochondrial dynamics and function change the size profiles of secreted vesicles	Enter stressed chondrocytes and incorporate into host mitochondrial networks	Donor cells express endogenous mitochondria-specific GFP (derived from PHaM mitoDendra2 mice) Recipient cell mitochondria labeled with MitoTracker Green	Thomas et al., 2022^59^
Astrocytes (rat)	Standard culture conditions Focal cerebral ischemia	Vesicles containing mitochondria	CD38/cADPR/calcium signaling	Enter neurons to promote neuronal survival	Donor cell mitochondria labeled with MitoTracker Red Recipient cell mitochondria labeled with CellLight Mitochondria-GFP	Hayakawa et al., 2016^26^
Astrocyte (mouse)	Antidepressant-like effect through the stimulation of sigma-1 receptor	Mitochondria (free or vesicle-enclosed not specified)	Increased CD38 expression	Support neuronal function	Donor cell mitochondria labeled with MitoTracker Red Recipient cells unlabeled	Wang et al., 2020^68^
Brain tissue (mouse, human) Fibroblasts (human)	Down syndrome Mitochondrial damage in vitro	Mitovesicles (EVs of mitochondrial origin)	Mitochondrial damage Mitophagy-independent	May serve as a biomarker to evaluate brain mitochondrial dysfunction in neurodegenerative disorders May eliminate detrimental mitochondrial components from the cell	Mitochondrial transfer NR	D’Acunzo et al., 2021^69^
Neural stem cells (mouse)	Standard culture conditions Multiple sclerosis (mouse model)	Vesicles containing mitochondria Free mitochondria	NR	Enter mononuclear phagocytes, fuse with the endogenous mitochondrial network, restore oxidative phosphorylation and reduce the expression of proinflammatory markers	Donor cells constitutively express the mitochondrial MitoDsRed reporter Recipient cell mitochondria stained with MitoTracker Green	Peruzzotti-Jametti et al., 2021^36^
Platelets (human)	Activated by thrombin	Microparticles containing mitochondria Free mitochondria	Actin polymerization independent of microtubules	Induce neutrophil proinflammatory responses through the generation of bioactive mediators (fatty acids, lysophospholipids, and mtDNA)	Donor mitochondria labeled with MitoTracker Deep Red Recipient cell mitochondria labeled with CellTracker CMTPX	Boudreau et al., 2014^16^
Platelets (human, mouse)	Activated Coculture with MSCs	Vesicles containing mitochondria Free mitochondria	NR	Enter MSCs and activate de novo fatty acid synthesis and trigger the secretion of pro-angiogenic factors	Donor mitochondria labeled with MitoTracker Green or isolated from C57BL/6J^su9-dsRed2^ transgenic mice that express RFP in mitochondria Recipient cells stained with WGA	Levoux et al., 2021^37^
Adipocytes (mouse, human)	Obesity	Vesicles containing oxidatively damaged mitochondria	nSMase pathway	Enter cardiomyocytes and induce transient oxidative stress in cardiac tissue, thereby triggering an antioxidant response	Donor cell mitochondria from mice that express a mitochondrion-localized Flag tag in adipocytes (adipo-mitoFlag mice) Recipient cardiomyocytes stained for cardiac troponin (CTN1)	Crewe et al., 2021^38^
Adipocytes (mouse)	Obesity	Not specified, but likely free or EV-associated mitochondria	NR	Enter macrophages through heparan sulfates to regulate metabolic homeostasis	Donor cell mitochondria from mice that express mitochondria-specific Dendra2 (mtD2 mice) Recipient macrophages labeled with CytoTracker Orange	Brestoff et al., 2021^61^
Brown adipocytes, BAT (mouse)	Thermogenic stress	Vesicles containing damaged mitochondrial parts/MDVs	PINK1-dependent efflux of mitochondrial proteins into EVs	Negatively affect thermogenesis through AMPK activation when taken up by brown adipocytes	Donor mitochondria labeled with MitoTracker Green Recipient brown adipocytes unlabeled	Rosina et al., 2022^14^
Adipocytes (mouse)	Lean Lard-HFD-induced obesity	Vesicles containing mitochondria Free mitochondria	NR	Enter macrophages and limit the release of adipocyte mitochondria into blood (lean) Circulate systemically and are distributed to distant organs (Lard-HFD-induced obesity)	Donor cell mitochondria from adipocyte-specific mitochondria reporter (MitoFat) mice Recipient cells unlabeled	Borcherding et al., 2022^60^
cFLIP-deficient MEFs Hepatocytes (mouse)	TNFα stimulation (in vitro) Anti-Fas antibody treatment (in vivo)	Free mitochondria	Mitochondrial fragmentation Actin and tubulin polymerization Caspase activation	Further investigation needed Possibly a source of antigens to trigger autoimmune diseases	Mitochondrial transfer NR	Nakajima et al., 2008^31^
Hepatocytes (rat) MEFs (mouse)	LPS stimulation	Autophagosomal membranes surrounding mitochondria/mitochondrial components	Extrusion via the autophagy‒lysosome pathway	Activate polymorphonuclear leukocytes	Direct mitochondrial transfer not shown	Unuma et al., 2015^70^
Hepatocytes (human)	Obesity	Microparticles containing mitochondria Free mitochondria (cellular origin unclear)	NR	Induce proinflammatory responses via TLR9 activation	Direct mitochondrial transfer not shown	Garcia-Martinez et al., 2016^42^
Hepatocytes (mouse)	Chronic-plus-binge ethanol feeding	Microparticles containing mtDNA/possibly mitochondria	ER stress-dependent caspase-1 activation	Activate TLR9 and neutrophilia, thereby causing liver inflammation and hepatocyte injury	Direct mitochondrial transfer not shown	Cai et al., 2017^71^
Cardiomyocytes (mouse)	Healthy Cardiac stress	Exophers containing defective mitochondria	Autophagy-driven	Enter cardiac-resident macrophages through the phagocytic receptor MERTK for elimination	Donor cardiomyocyte mitochondria labeled with mt-Keima through viral transduction Recipient macrophages stained for CD68	Nicolas-Avila et al., 2020^34^
Induced pluripotent stem cell-derived cardiomyocytes (human)	Short-term culture conditions	Vesicles containing mitochondria	NR	Improve mitochondrial bioenergetics in hypoxia-injured cardiomyocytes	Donor mitochondria labeled with BacMam mitochondria-RFP Recipient cell mitochondria with BacMam mitochondria-GFP	Ikeda et al., 2021^65^
C2C12 myotubes (mouse)	Iron deficiency	Vesicles containing mitochondria	Possible alternative to mitophagy Independent of BNIP3 and BNIP3l	Possibly an alternative or additional pathway to clear mitochondria under iron deprivation conditions Further investigation needed	Mitochondrial transfer NR	Leermakers et al., 2020^72^
Airway MDRCs (human)	Healthy Asthma	Vesicles/exosomes containing mitochondria	NR	Enter CD4 + T cells and generate reactive oxygen species	Donor mitochondria labeled with MitoTracker Green Recipient cells stained for CD4 or MitoTracker Red	Hough et al., 2018^39^
Endothelial progenitor cells (human)	Brain endothelial damage induced by OGD	Vesicles containing mitochondria	NR	Enter brain endothelial cells and improve brain endothelial energetics, barrier integrity, and angiogenic function	Donor cell mitochondria labeled with MitoTracker Red Recipient endothelial cells labeled with Rab5A-GFP	Hayakawa et al., 2018^15^
Osteoblasts (mouse)	Osteogenically differentiated	Vesicles containing mitochondria Free mitochondria	Mitochondrial fragmentation CD38/cADPR signaling	Promote the maturation of osteoprogenitors	Donor GFP+ mitochondria isolated from *Col1a1-Cre; Igs1*^*CKI-mitoGFP/+*^ osteoblasts Recipient cell mitochondria labeled with MitoTracker Red	Suh et al., 2023^27^
FADD-deficient Jurkat cell line (human) L929 fibroblast line (mouse)	TNF-α-induced necroptosis	Intact mitochondria	RIP1-dependent necroptosis	Enter macrophages, inducing the secretion of proinflammatory cytokines Enter dendritic cells to induce dendritic cell maturation	Donor mitochondria labeled with MitoTracker Green Recipient cells labeled with Phalloidin	Maeda & Fadeel, 2014^43^
THP-1 monocytic cell line (human)	LPS stimulation	Free mitochondria Microvesicles containing mitochondria	NR	Trigger proinflammatory responses in endothelial cells	Direct mitochondrial transfer not shown	Puhm et al., 2019^73^
PC12 cell line (rat) SH-SY5Y cell line HEK293 cell line HeLa cell line Skin fibroblasts (human)	Mitochondrial stress induction	Free mitochondria (majority) Vesicles containing mitochondria	Alternative to mitophagy	Act as an alternative pathway to mitophagy to clear damaged mitochondria	Mitochondrial transfer NR	Choong et al., 2020^33^
MDA-MB-231 cell line BT-549 cell line (human)	Chemoresistant	Exosomes containing mitochondria	nSMase pathway	Enter sensitive cancer cells and increase chemoresistance by increasing mutant mtDNA levels	Donor or recipient cells transfected with plasmids containing a mitochondrion-targeted sequence containing RFP or GFP inserts to observe bidirectional mitochondrial transfer	Abad & Lyakhovich, 2022^74^
High-metastatic (A11) and low-metastatic (P29) Lewis lung carcinoma cell lines (mouse)	Coculture system	Vesicles containing mitochondria/mitochondrial components	nSMase pathway	Enter low-metastatic cancer cells and stromal cells and possibly affect their metastatic ability and protumor activity, respectively Further investigation needed	Donor or recipient cell mitochondria labeled with MitoTracker Deep Red or CellLight mitochondria-GFP to observe bidirectional mitochondrial transfer	Takenaga et al., 2021^75^
HeLa cell line (human)	Mitochondrial stress induction	Free mitochondria	PINK1-Parkin-directed mitophagosome formation Autophagic secretion of mitochondria in the absence of mATG8-conjugation	Activate the cGAS-STING innate immune pathway in recipient cells	Direct mitochondrial transfer not shown	Tan et al., 2022^32^

*AdMSCs* adipose-derived mesenchymal stem cells (MSCs), *AMPK* adenosine monophosphate-activated protein kinase, *ARMMs* arrestin domain-containing protein 1 (ARRDC1)-mediated microvesicles, *BAT* brown adipose tissue, *BMSCs* bone marrow-derived MSCs, *BNIP3* BCL2-interacting protein 3, *BNIP3l* BNIP3-like, cADPR cyclic adenosine diphosphate (ADP)-ribose, *CD38* cluster of differentiation 38, *cFLIP* cellular FLICE-inhibitory protein, *cGAS* cyclic GMP-AMP synthase, *ER* endoplasmic reticulum, *EV* extracellular vesicle, *FADD* Fas-associated death domain, *GFP* green fluorescent protein, *HFD* high-fat diet, *HK-2* human kidney-2, *LPS* lipopolysaccharide, *MDRCs* myeloid-derived regulatory cells, *MDVs* mitochondrial-derived vesicles, *MEFs* mouse embryonic fibroblasts, *MERTK* tyrosine-protein kinase Mer, *MFN2* mitofusin 2, *mtDNA* mitochondrial DNA, *NR* not reported, *nSMase* neutral sphingomyelinase pathway, OGD oxygen-glucose deprivation, *PGC-1α* peroxisome proliferator activated receptor gamma coactivator 1 alpha, *RFP* red fluorescent protein, *RIP* receptor-interacting protein, *STING* stimulator of interferon genes, *TNF* tumor necrosis factor, *WGA* wheat germ agglutinin.

#### ARRDC1-mediated microvesicles

Extracellular release of mitochondria was reported as early as 2006^24^ in human MSCs under normal conditions in vitro. Vesicles containing fluorescently labelled mitochondria were released by MSCs onto tissue culture plates and contacted the plasma membranes of nearby cells^24^. Since then, a number of reports have demonstrated that human, mouse, and rat MSCs actively release microvesicles containing mitochondria into the extracellular space (Table 1). Regarding the mechanism of mitochondrial secretion, Phinney et al. showed that under standard culture conditions, MSCs manage oxidative stress by extracellularly releasing depolarized mitochondria through arrestin domain-containing protein 1 (ARRDC1)-mediated microvesicles (ARMMs)^25^. Live cell imaging showed that mitochondria travel toward the cell periphery and are included in the outward budding blebs of the plasma membrane^25^. Electron microscopy confirmed the presence of microvesicles containing mitochondria in MSC-conditioned media^25^.

#### CD38/cADPR/calcium signaling

In addition to MSCs, astrocytes have been shown to secrete mitochondria into the extracellular space through a calcium-dependent mechanism (Table 1). In 2016, Hayakawa et al. reported the presence of extracellular particles (0.3-1.1 μm in diameter) containing functional mitochondria released from rat cortical astrocytes^26^. A high percentage of extracellular mitochondria-containing particles were β1-integrin- and CD63-positive and were released via CD38/cyclic ADP-ribose (cADPR)/calcium signaling in astrocytes^26^. Similarly, our group recently demonstrated that mature osteoblasts secrete CD63-positive mitochondria-containing vesicles (>0.2 μm in diameter) into the extracellular space partly through CD38/cADPR signaling^27^. CD38 is highly expressed in osteoblasts^28^ and has been suggested to play a critical role during bone formation, and *Cd38*-knockout mice exhibit an osteoporotic phenotype^29,30^. Our group showed that the *Cd38* expression pattern in differentiating osteoblasts coincided with the pattern of mitochondrial secretion, and knockdown of *Cd38* significantly impaired mitochondrial release^27^. However, whether CD38/cADPR signaling is specific to mitochondrial release or regulates mitochondrial secretion by other cell types requires further investigation.

#### Actin polymerization

Activated platelets can release functional mitochondria into the extracellular space through actin dynamics (Table 1). Boudreau et al. demonstrated that intact free mitochondria or microparticles containing mitochondria were present in the supernatant of thrombin-activated human platelets^16^. The group used actin polymerization inhibitors (cytochalasin B, D, E, or latrunculin A) or tubulin polymerization inhibitor (nocodazole) and found that the extrusion of free mitochondria and microparticles containing mitochondria was significantly decreased by the addition of actin inhibitors but not the tubulin inhibitor, suggesting that mitochondrial secretion requires intact actin but not microtubule dynamics^16^. However, the release of microparticles without mitochondria also significantly decreased in response to the actin inhibitors and not the tubulin inhibitor, suggesting that the mechanism may not be mitochondria-specific but may apply to microparticle secretion in general. The involvement of actin polymerization in mitochondrial extrusion was also demonstrated in cellular FLICE-like inhibitory protein (cFLIP)-deficient mouse embryonic fibroblasts (MEFs) stimulated with tumor necrosis factor alpha (TNF-α)^31^. Unlike in platelets, exposure to an actin polymerization inhibitor (cytochalasin D) or tubulin destabilizer (paclitaxel) impaired cytoplasmic vacuole formation and the subsequent secretion of free mitochondria by MEFs, indicating that both intact actin and tubulin dynamics are essential for mitochondrial release by MEFs^31^.

#### Changes in mitochondrial morphology

Specific alterations in mitochondrial morphology have been suggested as a mechanism leading to extracellular mitochondrial secretion^27,31^. Nakajima et al. showed that cytoplasmic vacuoles within cFLIP-deficient MEFs engulfed fragmented mitochondria but not elongated mitochondria and released them into the extracellular space in response to TNF-α stimulation, indicating that mitochondrial fragmentation is a prerequisite for their extracellular release^31^. Likewise, our group recently reported that mitochondrial fragmentation and donut formation, which actively produce MDVs, stimulated mitochondrial extrusion from osteoblasts^27^. We demonstrated that inducing mitochondrial fission and donut formation by knocking down *Opa1*, which mediates mitochondrial fusion, or overexpressing *Fis1*, which promotes mitochondrial fission and MDV formation^8^, significantly increased the extracellular release of mitochondria, while treatment with the mitochondrial fusion promoter M1 prevented mitochondrial secretion by osteoblasts^27^. These results indicate that mitochondrial dynamics may play direct and critical roles in mediating mitochondrial extrusion. Close examination of mitochondrial morphology in different cell types that secrete mitochondria will help determine whether this mechanism applies universally.

#### Secretory autophagy

Extracellular release of mitochondria may be a mitochondrial quality control (MQC) process alternative to mitophagy^32–34^. Nicolas-Avila et al. reported that healthy or stressed mouse cardiomyocytes ejected defective mitochondria into the extracellular space through LC3-positive membrane vesicles called exophers, which are distinct from classical EVs in that they are larger in size (3.5 $\pm$ 0.1 μm in mean diameter), contain large organelles such as mitochondria and are driven by the autophagy machinery^34^. The group showed that extracellular secretion of damaged mitochondria through exophers was a mechanism of mitochondrial quality control^34^. In the same year, Choong et al. suggested that extracellular release of mitochondria was an alternative MQC system to maintain mitochondrial homeostasis in rat PC12 cells and several human cell lines^33^. In support of this conclusion, genetic deletion or knockdown of autophagy/mitophagy genes significantly increased mitochondrial secretion into the extracellular environment through direct budding from the plasma membrane to compensate for defective mitophagy^33^. Likewise, Tan et al. reported that during PINK1-Parkin-mediated mitophagy, damaged mitochondria could still be cleared (through extracellular secretion) without the mammalian ATG8 (mATG8)-conjugation system, which is a crucial step in autophagy that leads to lysosomal degradation^32^. The group suggested that mitochondria were extruded through the secretory autophagy pathway that releases secretory cargos within autophagosomes, which was supported by data showing that genetic inhibition of autophagosome formation or autophagosome-plasma membrane fusion decreased mitochondrial secretion^32^. Through biochemical analysis and proteinase K protection analysis of the EV fraction of ATG7-knockout HeLa cells, the group demonstrated that secreted mitochondria were present as free organelles and were not enclosed by EVs^32^. Overall, these findings indicate that different mechanisms of mitochondrial secretion exist in a variety of cell types. However, further investigation is necessary to determine whether the various mechanisms discussed above act in conjunction with each other or if there is a distinct molecular mechanism that is specific to the secretion of mitochondria other than general EV secretion.

### Biological effects of extracellular mitochondria on target cells

Extracellularly secreted mitochondria have been shown to target recipient cells to modulate various metabolic, immune, or differentiation/maturation responses (Table 1). In the following section, we review the major biological effects of secreted mitochondria on recipient cells.

#### Metabolic effects

The most widely reported biological effects of extracellular mitochondria are their incorporation into recipient cells and subsequent regulation of metabolism, such as mitochondrial bioenergetics and the oxidative stress response. For instance, extracellular mitochondria secreted by MSCs are taken up by macrophages through phagocytosis and fuse with host mitochondria to enhance the macrophage oxygen consumption rate (mitochondrial respiration)^25^. MSC-derived extracellular mitochondria are also internalized by IEC-6 intestinal epithelial cells and fused with host mitochondria to improve mitochondrial function^35^. Hayakawa and colleagues demonstrated that extracellular mitochondria secreted by astrocytes entered neurons and enhanced intracellular ATP levels and neuronal viability^26^. Similarly, mitochondria extruded by neural stem cells were taken up by mononuclear phagocytes by endocytosis and integrated into the endogenous mitochondrial network to restore oxidative phosphorylation^36^. Furthermore, activated platelets secrete functional mitochondria that are subsequently incorporated into MSCs to stimulate the tricarboxylic acid (TCA) cycle and de novo fatty acid synthesis, thereby triggering the secretion of proangiogenic factors^37^. In addition to regulating mitochondrial bioenergetics in recipient cells, extracellular mitochondria have been shown to regulate the oxidative stress response. Small EVs containing oxidatively damaged mitochondria released by adipocytes were internalized into cardiomyocytes to induce a burst of reactive oxygen species (ROS), triggering a compensatory antioxidant response and protecting cardiomyocytes through hormesis^38^. Likewise, exosomes containing polarized mitochondria (MitoTracker Green-positive) secreted by proinflammatory human leukocyte antigen-antigen D related (HLA-DR)-positive airway myeloid-derived regulatory cells (MDRCs) were taken up by peripheral T cells, and MitoTracker Green-positive mitochondria were integrated into the host mitochondrial network, possibly affecting T-cell differentiation and function through ROS generation^39^.

#### Anti- or proinflammatory effects

In addition to modulating metabolism, extracellular mitochondria have been shown to trigger anti- or proinflammatory responses in recipient cells. The transfer of EVs containing mitochondria derived from MSCs to human macrophages enhanced phagocytosis and downregulated proinflammatory cytokine secretion by macrophages^40^. Similarly, mitochondria and mitochondrial DNA (mtDNA) transfer through exosomes released by adipose-derived MSCs restored mitochondrial integrity in macrophages and induced their shift to an anti-inflammatory phenotype by suppressing proinflammatory cytokine (IL-1β and TNF-α) secretion and upregulating anti-inflammatory cytokine (IL-10 and Arg-1) production^41^. The expression of proinflammatory genes (*Il1β*, *Nos2*, and *Il6*) was also significantly downregulated in mononuclear phagocytes in response to extracellular mitochondria released by neural stem cells^36^. Although further research is necessary, the reported anti-inflammatory effects of extracellular mitochondria appear to be secondary responses to improvements in mitochondrial integrity and function.

Secreted mitochondria have also been reported to stimulate proinflammatory responses in recipient cells. For example, extracellular mitochondria released by activated platelets were shown to act as endogenous substrates of secreted phospholipase A_2_ IIA (sPLA_2_-IIA), which hydrolyzes the mitochondrial membrane and leads to the generation of inflammatory mediators such as lysophospholipids, fatty acids, and mtDNAs that induce a proinflammatory response in neutrophils^16^. Furthermore, extracellular mitochondria or mtDNA enclosed by microparticles released from hepatocytes activate Toll-like receptor 9 (TLR9) and the proinflammatory response in lysozyme-expressing cells such as neutrophils, monocytes, and macrophages^42^. Damaged mitochondria released extracellularly by HeLa cells via the secretory autophagy pathway induced a proinflammatory response in recipient HeLa cells by activating the cGAS-STING pathway, possibly through mtDNA^32^. Based on these reports, mtDNA, which is a well-known and potent damage-associated molecular pattern (DAMP), appears to be largely responsible for the proinflammatory response triggered by extracellular mitochondria. Importantly, specific mechanisms that induce or prevent the proinflammatory effects of mtDNAs on recipient cells warrant further investigation.

#### Cell differentiation/maturation effects

Extracellular mitochondria have been shown to regulate target cell differentiation or maturation. Recently, Rosina and colleagues reported that brown adipocytes released EVs containing MDVs with damaged mitochondrial parts, which were incorporated into recipient brown adipocytes and activated AMP-activated protein kinase (AMPK) to suppress adipocyte differentiation and thermogenic potential by downregulating the expression of *Cd36*, *Fabp4*, and *Ucp1*^14^. The group suggested that oxidized materials and high levels of AMP contained within EVs could trigger AMPK in target cells to prevent adipogenesis^14^. Regarding the effects of extracellularly secreted mitochondria on stimulating target cell maturation, mitochondria released by necroptotic, Fas-associated protein with death domain (FADD)-deficient Jurkat cells were engulfed by human monocyte-derived dendritic cells (MDDCs) and promoted their maturation, inducing the cell surface markers CD80, CD83, and CD86^43^. Furthermore, our group recently reported that mitochondria secreted from mature osteoblasts enhanced the osteogenic maturation of osteoprogenitor cells without affecting mitochondrial respiration through the delivery of cargo proteins^27^. We showed that the incorporation of intact extracellular mitochondria into recipient cells was not required for this effect as treatment with secreted mitochondria after a repeated freeze/thaw cycle did not abrogate the increase in the maturation of osteoprogenitors. Instead, exposure of extracellular mitochondria to proteinase K abolished their ability to stimulate osteoprogenitor maturation, indicating that specific proteins within mitochondria were responsible for this effect. Whether the proteins activate surface receptors or are taken up by target cells needs further investigation, but the results suggest a mechanism that does not involve direct metabolic effects mediated by the integration of intact mitochondria into recipient cells^27^.

## Mitochondrial transplantation therapy

### Therapeutic effects of mitochondrial transplantation on animal models

The beneficial effects of extracellularly secreted mitochondria on the metabolism and function of recipient cells indicate that exogenous supplementation with mitochondria could induce curative responses through similar mechanisms. As with those secreted extracellularly by donor cells, injected mitochondria have been shown to enter recipient cells to regulate target cell metabolism, inflammatory response, or their differentiation/maturation in vivo (Table 2). Through these effects, mitochondrial transplantation has been shown to repair damaged tissues, including but not limited to the heart, brain, spinal cord, liver, lungs, kidney, musculoskeletal tissues, and intestine, in animal models of critical illnesses (Table 2). Currently, several clinical trials are recruiting or selecting patients to investigate the effects of mitochondrial treatment on myocardial ischemia, cerebral ischemia, or inflammatory muscle diseases such as polymyositis and dermatomyositis (Table 3). Thus, we will briefly review the major therapeutic effects of mitochondrial transplantation on animal models of cardiac ischemia, cerebral ischemia, and decline in skeletal muscle function or mass. Descriptions of mitochondrial transplantation in animal models of other diseases are provided in Table 2.

**Table 2:** Mitochondrial transfer in animal models.

Tissue	Disease/Injury (animal model)	Intervention	Mitochondrial source	Mitochondrial target/distribution	Target/uptake mechanism	Effects	Reference
Heart	Cardiac ischemia (Rabbits subjected to regional ischemia)	Injection of mitochondria directly into the ischemic zone	Nonischemic heart	Interfibrillar space near the epicardial surface	Distribution aided by interfibrillar separation after myocardial ischemia and myocardial contraction	Improved regional and global function recovery	McCully et al., 2009^44^
			Autologous pectoralis major muscle	Cardiomyocytes	Internalization within hours of transplantation Possible extracellular effects without internalization Viable mitochondria required	Increased oxygen consumption rate, ATP production, and cardioprotective cytokine induction Enhanced postinfarct cardiac function	Masuzawa et al., 2013^45^
	Cardiac ischemia (Rabbits subjected to regional/global ischemia)	Infusion of mitochondria through the coronary artery (less invasive than direct injection into the ischemic zone)	Autologous liver	Widespread myocardial dispersion	Rapid widespread distribution of mitochondria throughout the myocardium	Reduced infarct size Enhanced cardiac function in regionally ischemic hearts	Cowan et al., 2016^76^
	Cardiac ischemia (Pigs subjected to regional ischemia)	Injection of mitochondria into the ischemic area	Autologous pectoralis major muscle	Cardiomyocytes	Internalization possibly through actin-dependent endocytosis	Decreased infarct size Enhanced myocardial cell viability	Kaza et al., 2017^46^
	Heterotopic heart transplantation (Mice subjected to heart transplantation)	Injection of mitochondria into the coronary ostium	Gastrocnemius muscle	Heart	Diffuse distribution	Prolonged cold ischemia time Decreased neutrophil infiltration and necrosis Enhanced heart graft tissue viability and function	Moskowitzova et al., 2019^77^
	Cardiac ischemia (Rats subjected to warm global ischemia)	Administration of mitochondria to the coronary artery through an aortic cannula	Autologous pectoralis major muscle	Heart tissue Myocardial fibers	Internalization possibly through actin-dependent endocytosis	Increased myocardial function and myocellular survival in diabetic heart	Doulamis et al., 2020^47^
	Cardiac ischemia (Pigs subjected to regional ischemia)	Preischemic intracoronary injection of mitochondria	Autologous pectoralis major muscle	Cardiac cells	Internalization possibly through actin-dependent endocytosis	Increased global and regional myocardial function Decreased infarct size	Guariento et al., 2020^78^
	Right heart failure (Piglets subjected to pulmonary artery banding)	Intramyocardial injection of mitochondria	Autologous gastrocnemius muscle	Cardiomyocytes	Internalization of viable mitochondria	Preserved contractile function Decreased cardiomyocyte apoptosis and fibrosis	Weixler et al., 2021^79^
	Myocardial infarction (Mice subjected to surgical ligation of LAD)	Intramyocardial injection of mitochondria-rich EVs	EVs from human iCM	Cardiomyocytes	Fusion of the EV lipid bilayer with the cell membrane to release mitochondria into the cytoplasm	Improved bioenergetics and mitochondrial biogenesis in the peri-infarct area	Ikeda et al., 2021^65^
		Retro-orbital injection of small EVs containing mitochondria	Small EVs from adipocytes	Cardiomyocytes	Integration into the host mitochondrial network	Decreased infarct size Reduced cardiac hypertrophy Increased cardiac function	Crewe et al., 2021^38^
	Heart donated after circulatory death (Pediatric and neonatal pigs)	Ex situ heart perfusion of mitochondria	Autologous skeletal muscle	Heart	Not specified A mechanism previously reported by the group	Improved myocardial function and viability	Alemany et al., 2023^80^
	Sepsis (Rats subjected to cecal ligation and puncture procedure)	Tail vein injection of mitochondria	Pectoralis major muscle	Heart	Internalization into heart tissue	Improved survival rate Enhanced mitochondrial function and biogenesis in the heart	Mokhtari et al., 2023^81^
Brain	Cerebral ischemia (Rats subjected to MCAO)	Injection of mitochondria into the ischemic striatum Infusion of mitochondria into the femoral artery	BHK-21 cell line	Neurons Astrocytes Microglia	Internalization may not be solely responsible for the effect Intact mitochondria required	Restored motor performance Reduced infarct area and neuronal cell death	Huang et al., 2016^48^
		Intravenous injection of multipotent MSCs	Multipotent MSCs	Neural cells	Mitochondrial transfer through Miro 1-mediated TNTs	Improved recovery of neurological functions	Babenko et al., 2018^82^
		Intracerebroventricular injection of mitochondria	Autologous pectoralis major muscle	Neurons	Distribution and internalization in penumbra areas	Decreased oxidative stress and cell death Increased neurogenesis Decreased infarct volume	Zhang et al., 2019^49^
		Injection of mitochondria into the internal carotid artery	Neuro-2a cell line Mouse neural stem cell	Unspecified Mitochondria could not pass the blood‒brain barrier	Internalization into host cells and possible separation of mitochondrial components	Improved neurobehavioral deficits Reduced infarct size	Xie et al., 2021^52^
		Intracerebroventricular injection of mitochondria	Human umbilical cord-derived MSCs	Brain cells (possibly neurons and astrocytes)	Internalization into recipient cells	Decreased infarct area Improved neurobehavioral functions	Pourmohammadi-Bejarpasi et al., 2020^83^
		Injection of mitochondria into the ischemic striatum	Astrocytes	Neurons	Internalization into recipient cells	Decreased infarct size	Lee et al., 2023^51^
	Cerebral ischemia (Mice subjected to MCAO)	Intravenous infusion of mitochondria	Placenta	Brain Peripheral organs (lung, liver, kidney, heart)	Further investigation needed	Decreased infarct size	Nakamura et al., 2020^50^
	Traumatic brain injury (Rats subjected to weight-drop injury)	Stereotaxic injection of mitochondria into the right lateral ventricle	Human umbilical cord-derived MSCs	Brain cells	Internalization of intact mitochondria	Improved motor function Increased brain cell survival Alleviated astrogliosis and microglia activation	Bamshad et al., 2023^84^
	Parkinson’s disease (6-hydroxydopamine-induced rats)	Injection of mitochondria into the medial forebrain bundle	PC12 cell line (allogeneic) Human osteosarcoma cybrids (xenogeneic)	Neurons	Internalization into recipient cells	Improved locomotion Decreased oxidative stress and dopaminergic neuron loss	Chang et al., 2016^85^
		Intranasal infusion of mitochondria	Liver	Rostral migratory stream neurons	Internalization into recipient cells	Improved rotational and locomotor behaviors and neuronal survival Decreased plasma inflammatory cytokine levels	Chang et al., 2021^86^
	Alzheimer’s disease (Amyloid-beta-injected mice)	Intravenous injection of mitochondria	HeLa DsRed2-mito cells	Liver Possibly the brain	Further investigation needed	Improved cognitive performance Decreased neuronal loss and gliosis Increased liver mitochondrial activity	Nitzan et al., 2019^87^
	Depression (LPS-injected mice)	Intravenous injection of mitochondria	Hippocampus	Possibly brain cells	Further investigation needed	Attenuated depression-like behaviors Decreased oxidative stress and neuroinflammation	Wang et al., 2019^88^
	Age-associated cognitive decline (Aged mice)	Tail vein injection of mitochondria	Liver of young mice	Possibly the brain and skeletal muscle	Further investigation needed	Improved cognitive and motor performance	Zhao et al., 2020^53^
		Injection of mitochondria into the hippocampus	Liver of young mice	Neural progenitors	Internalization into recipient cells	Improved cognitive performance Enhanced neurogenesis	Zhang et al., 2022^89^
Spinal cord	Spinal cord injury (Rats subjected to contusion)	Injection of mitochondria into the spinal cord	PC12 cell line Soleus	Brain macrophages, endothelial cells, pericytes, astrocytes, and oligodendrocytes	Internalization into recipient cells May exert their effects extracellularly	Partial recovery of acute bioenergetics No improvement in long-term functional recovery Further investigation needed	Gollihue et al., 2018^90^
			BMSCs	Neurons, astrocytes, macrophages	Internalization into recipient cells BMSCs transfer mitochondria to neurons through gap junctions	Enhanced locomotor functional recovery	Li et al., 2019^91^
	Spinal cord injury (Rats subjected to compression)	Transplantation of mitochondria into the spinal cord using a microsyringe pump	Soleus	Extracellular spaces	Interstitial localization rather than internalization into recipient cells	Improved locomotor functions Decreased mitochondrial fragmentation, apoptosis, inflammation, and oxidative stress	Lin et al., 2022^92^
Liver	Partial liver ischemia (Rats subjected to partial hepatic I/R injury)	Splenic injection of mitochondria	Liver	Hepatocytes	Internalization of intact mitochondria	Decreased serum aminotransferase level Decreased hepatocyte death, oxidative stress, and tissue injury	Lin et al., 2013^93^
	Nonalcoholic fatty liver disease (Mice subjected to high-fat and high-cholesterol diet)	Intravenous injection of mitochondria	HepG2 cell line	Liver, lung, brain, muscle, and kidney	Internalization into various tissue cells	Decreased serum aminotransferase and cholesterol levels Decreased lipid accumulation Increased energy production and hepatocyte function	Fu et al., 2017^94^
	Acetaminophen-induced liver injury (Acetaminophen-injected mice)	Intravenous injection of mitochondria	HepG2 cell line	Hepatocytes, brain, lung, kidney, muscle	Internalization through endocytosis	Increased energy supply Decreased oxidative stress and tissue injury	Shi et al., 2018^95^
	Acetaminophen-induced liver injury (Acetaminophen-administered rats)	Injection of mitochondria into the subcapsular region of spleen	Rat MSC cell line	Hepatocytes	Internalization of intact mitochondria	Improved liver structure Decreased serum aminotransferase level, cell death, and oxidative stress	Ulger et al., 2021^96^
	Liver ischemia (Mice subjected to liver I/R injury)	Intravenous injection of EVs containing mitochondria	EVs from human umbilical cord-derived MSCs	Intrahepatic neutrophils	Internalization of functional mitochondria	Improved liver I/R injury Decreased local neutrophil extracellular trap formation	Lu et al., 2022^97^
Lungs	Acute lung injury (LPS-induced mice)	Intranasal instillation of wild-type BMSCs	BMSCs	Alveolar epithelial cells	Mitochondria-containing microvesicles through connexin 43-containing gap junctional channels	Increased alveolar ATP concentrations Increased mouse survival	Islam et al., 2012^98^
		Tail vein injection of exosomes containing mitochondrial components	Exosomes from AdMSCs	Alveolar macrophages	Exosomes containing mitochondria and mtDNA Internalized through clathrin and caveolae-mediated endocytosis	Alleviated lung damage through decreased inflammation and alveolar wall thinning Improved mitochondrial function and immune homeostasis of alveolar macrophages	Xia et al., 2022^41^
		Intratracheal injection of MSCs	MSCs	PMVECs	Mitochondrial transfer through TFAM-mediated TNTs	Alleviated LPS-induced lung edema and improved permeability barrier of PMVECs	Zhang et al., 2023^99^
	Acute lung injury (Ischemia‒reperfusion injury-induced mice)	Pulmonary artery injection or aerosol delivery via the trachea (nebulization) of mitochondria	Gastrocnemius muscle	Lung alveoli and connective tissue	Not specified Possible internalization through actin-dependent endocytosis	Improved lung mechanics and decreased lung tissue injury	Moskowitzova et al., 2020^100^
	Pulmonary fibrosis (Bleomycin-treated mice)	Tail vein injection of hMSCs or mitochondria isolated from hMSCs	hMSCs	Alveolar epithelial cells	Mitochondrial transfer through connexin 43-containing gap junctional channels	Mitigation of fibrotic progression through restoring mitochondrial function	Huang et al., 2021^101^
Kidney	Renal artery stenosis (Mice subjected to surgical placement of arterial cuff)	Intra-arterial injection of EVs containing mitochondria	EVs from STC-like cells isolated from pig kidneys	Tubular epithelial cells	Internalization of viable mitochondria	Improved kidney perfusion and oxygenation Decreased fibrosis	Zou et al., 2018^102^
	Diabetic nephropathy (Streptozotocin-induced rats)	Injection of mitochondria directly under the renal capsule	BMSCs	Proximal tubular epithelial cells Renal tubules	Internalization into recipient cells	Improved histology of proximal tubules Improved structure of tubular basement membrane and brush border	Konari et al., 2019^103^
	Acute kidney injury (Rats subjected to renal I/R injury)	Injection of mitochondria into the renal artery	Pectoralis major muscle	Kidney	Further investigation needed	Increased regenerative potential of renal cells Decreased renal cell death	Jabbari et al., 2020^104^
	Acute kidney injury (Pigs subjected to renal I/R injury)	Intra-arterial injection of mitochondria	Sternocleidomastoid muscle	Kidney Tubular epithelium of cortex and medulla	Internalization possibly through actin-dependent endocytosis	Increased renal function and tissue recovery	Doulamis et al., 2020^105^
	Acute kidney injury (Mice subjected to renal I/R injury)	Tail vein injection of EVs containing mitochondrial components	EVs from BMSCs	Liver, lung, kidney, and spleen	Internalization into renal tubular epithelial cells and recover TFAM protein and mtDNA stability	Attenuated renal dysfunction, mitochondrial damage, and inflammatory response	Zhao et al., 2021^106^
	Diabetic nephropathy (Streptozotocin-induced mice)	Tail vein injection of mitochondria-transferred macrophages	MSCs	Macrophages (artificially transferred in vitro)	Internalization into recipient cells (artificially transferred in vitro)	Suppressed inflammatory response Improved kidney injury	Yuan et al., 2021^107^
Skeletal muscle	Age-associate decline in muscle function (Aged mice)	Tail vein injection of mitochondria	Liver of young mice	Possibly brain and skeletal muscle	Further investigation needed	Enhanced skeletal muscle function (swim and rotarod test)	Zhao et al., 2020^53^
	Acute limb ischemia (Tourniquet-applied mice)	Injection of mitochondria into hindlimb muscles	Gastrocnemius or vastus medialis muscles	Myofibers in soleus, vastus medialis, and gastrocnemius	Internalization into recipient cells	Decreased infarct size Enhanced hindlimb function	Orfany et al., 2020^54^
	Muscle injury (BaCl_2_-injected mice)	Injection of mitochondria-transferred C2C12 cells into the gastrocnemius muscle	C2C12 cell line	C2C12 cell line (artificially transferred in vitro)	Internalization via endocytosis (artificially transferred in vitro)	Improved muscle regeneration and function in response to injection of mitochondria-transferred C2C12 cells	Sun et al., 2022^108^
		Tail vein injection of mitochondria	Liver	Damaged myofibers	Internalization of intact mitochondria (possibly through damaged myofiber membrane)	Improved muscle regeneration Restored muscle function	Alway et al., 2023^55^
	Muscle atrophy (Dexamethasone-injected rats)	Injection of mitochondria into soleus muscles	Umbilical cord-derived MSCs	Soleus muscle	Possible internalization into muscle-resident cells Further investigation needed	Increased muscle mass	Kim et al., 2023^56^
Bone/Cartilage	Bone defect (Rats subjected to cranial defect surgery)	Local treatment of mitochondria-transferred BMSCs	BMSCs	BMSCs (artificially transferred in vitro)	Internalization into recipient cells (artificially transferred in vitro)	Enhanced bone regeneration due to increased osteogenic activity of BMSCs	Guo et al., 2020^109^
	Osteoarthritis (Monosodium iodoacetate-induced rats)	Intra-articular injection of mitochondria into the right knee	L6 myoblast cell line	Chondrocytes (in vitro)	Internalized into recipient cells (in vitro)	Improved pain, cartilage destruction, and bone loss	Lee et al., 2022^110^
	Bone defects (Mice subjected to cranial defect surgery)	Local treatment with extracellular mitochondria	Extracellular mitochondria released from mature osteoblasts	Osteoprogenitors (in vitro)	Effect mediated by protein cargo within mitochondria (in vitro) Intact mitochondria may be unnecessary	Enhanced bone regeneration	Suh et al., 2023^27^
Intestine	Sepsis (Rats subjected to cecal ligation and puncture procedure)	Intravenous injection of vesicles containing mitochondria/mitochondrial proteins	Extracellular microvesicles from MSCs	Intestinal epithelial cells	Internalization into recipient cells and delivery of mitochondrial proteins, promoting mitochondrial fusion and biogenesis	Improved mitochondrial function and intestinal barrier function	Zheng et al., 2021^35^

*AdMSCs* adipose-derived mesenchymal stem cells, *BaCl*_*2*_ barium chloride, *BMSCs* bone marrow-derived MSCs, *EV* extracellular vesicle, *hMSCs* human MSCs, *iCM* induced pluripotent stem cell-derived cardiomyocytes, *I/R* ischemia/reperfusion, *LAD* left anterior descending coronary artery, *LPS* lipopolysaccharide, *MCAO* middle cerebral artery occlusion, *mtDNA* mitochondrial DNA, *PC12* adrenal pheochromocytoma, *PMVECs* pulmonary microvascular endothelial cells, *STCs* scattered tubular cells, *TFAM* transcription factor A, mitochondrial, *TNTs* tunneling nanotubes.

**Table 3:** Clinical trials involving mitochondrial transplantation.

Conditions	Interventions	Location	Mitochondria donor	Status	Year	NCT number
Low ovarian reserve Poor quality oocytes	Injection of mitochondria into oocytes	Hadassah University Hospital, Israel	Autologous granulosa cells	Withdrawn (phase 1/2)	Start: 2012 Primary completion: 2015 Study completion: 2015	NCT01631578
Infertility	Microinjection of mitochondria into oocytes as a complementary ICSI technique	IVI Valencia, Spain	Autologous ovarian stem cells	Completed (phase NA)	Start: 2015 Primary completion: 2017 Study completion: 2017	NCT02586298
Extracorporeal membrane oxygenation complication	Injection or infusion of mitochondria into the ischemic myocardium	Boston Children’s Hospital, USA	Autologous skeletal muscle of the chest wall	Recruiting (phase NA)	Start: 2017 Estimated primary completion: 2024 Estimated study completion: 2025	NCT02851758
Repetition failure	Injection of mitochondria into oocytes	Location not specified Sponsored by Sun Yat-sen University	BMSCs	Unknown	Estimated start: 2018 Estimated primary completion: 2020 Estimated study completion: 2021	NCT03639506
Mitochondrial diseases Pearson syndrome	Transplantation of mitochondria-transplanted hematopoietic stem cells	Sheba Medical Center Hospital-Tel Hashomer, Israel	Normal and healthy blood cells	Completed (phase 1/2)	Start: 2019 Primary completion: 2021 Study completion: 2021	NCT03384420
Cerebral ischemia	Infusion of mitochondria into the brain artery via microcatheter during reperfusion	Harborview Medical Center, USA	Autologous muscle tissue adjacent to the surgical access site	Recruiting (phase 1)	Start: 2021 Estimated primary completion: 2024 Estimated study completion: 2024	NCT04998357
Polymyositis Dermatomyositis	Intravenous administration of a single-dose of allogeneic mitochondria (PN-101)	Seoul National University, Korea Soonchunhyang University Seoul Hospital, Korea Hanyang University Seoul Hospital, Korea	Allogeneic umbilical cord-derived mesenchymal stem cells	Enrolling by invitation (phase 1/2)	Start: 2021 Estimated primary completion: 2023 Estimated study completion: 2023	NCT04976140
Myocardial infarction Myocardial ischemia Myocardial stunning	Intracoronary and intramyocardial injection of mitochondria and/or MSC-derived exosomes	Tehran University of Medical Sciences, Iran	MSCs for exosomes and autologous tissues for mitochondria	Recruiting (phase 1/2)	Start: 2022 Estimated primary completion: 2023 Estimated study completion: 2024	NCT05669144

*BMSC* bone marrow-derived mesenchymal stem cell, *ICSI* intracytoplasmic sperm injection, *NA* not applicable.

#### Cardiac ischemia

The first therapeutic evaluation of mitochondrial transplantation in an animal model dates back to 2009 when McCully et al. demonstrated that directly injecting viable mitochondria isolated from a nonischemic heart into the ischemic zone of cardiac tissue promoted myocardial functional recovery and cell viability in rabbits subjected to ischemia/reperfusion (I/R) injury^44^. The authors emphasized that the isolated mitochondria must be fresh, viable, and respiration-competent to induce cardioprotective effects, since frozen mitochondria or separated mitochondrial components failed to do so^44^. Since then, McCully’s team has actively investigated mitochondrial transplantation therapy for cardiac ischemia in rabbits, pigs, and rats (Table 2). Masuzawa et al. reported that injecting mitochondria isolated from autologous skeletal muscle into the ischemic heart attenuated myocardial injury and improved cardiac function in rabbits subjected to I/R injury^45^. The group showed that the injected mitochondria were taken up by cardiomyocytes 2 hours after administration, and this uptake resulted in an increase in the oxygen consumption rate and ATP production, while also leading to a significant decrease in serum inflammatory markers^45^. Notably, the authors suggested that the transplanted mitochondria also exerted extracellular effects (without internalization by cardiomyocytes) as cardioprotection was apparent within 10 minutes of reperfusion when mitochondria were present in the interstitial space outside of cardiomyocytes^45^. The authors also highlighted the importance of transplanting viable mitochondria^45^. A few years later, McCully’s team reported similar findings in a porcine model of I/R, suggesting that injecting mitochondria derived from autologous skeletal muscle into the ischemic area of cardiac tissue improved myocardial cell viability^46^, and recently reported the cardioprotective effects of mitochondrial transplantation on diabetic rat hearts subject to warm global ischemia^47^.

#### Cerebral ischemia

Mitochondrial transplantation has also exerted beneficial effects on animal models of cerebral ischemia. In 2016, Huang et al. first demonstrated that local intracerebral or systemic intra-arterial injection of xenogenic hamster mitochondria (isolated from the BHK-21 hamster cell line) significantly rescued motor performance and attenuated infarct size and neuronal cell death in a rat ischemic stroke model induced by middle central artery occlusion (MCAO)^48^. The group detected the internalization of transplanted mitochondria into neurons and astrocytes but with low efficacy, suggesting that cellular uptake of exogenous mitochondria may not be necessary for their neuroprotective effects^48^. A few years later, Zhang et al. used the same rat stroke model of MCAO and showed that intracerebroventricular (ICV) injection of mitochondria isolated from autologous skeletal muscle led to their internalization by neurons, especially in the ischemic penumbra, thereby increasing ATP levels and neurogenesis while decreasing oxidative stress, apoptosis, reactive astrogliosis, and brain infarct volume^49^. Nakamura et al. also showed that in a mouse MCAO model, intravenous infusion of mitochondria isolated from cryopreserved placentas significantly reduced infarction volume^50^. The infused mitochondria crossed the blood‒brain barrier and were distributed in the ischemic brain and peripheral organs such as the lung, liver, kidney, and heart, the effects of which are yet to be determined^50^. Xie et al. and Lee et al. further confirmed the therapeutic potential of mitochondrial transplantation, which decreased infarct volume and improved cell survival in a rat MCAO model of stroke^51,52^.

#### Loss of skeletal muscle function or mass

The therapeutic effects of mitochondrial transplantation on skeletal muscle tissues have only recently been investigated. In 2020, Zhao et al. demonstrated that tail vein injection of mitochondria isolated from young mouse livers significantly improved skeletal muscle function in aged mice (18-month-old mice with compromised muscle function), which was assessed by the forced swimming test for muscular endurance and rotarod test for muscular coordination^53^. Mitochondrial treatment significantly increased pyruvate dehydrogenase, α-ketoglutarate dehydrogenase, NADH dehydrogenase, and ATP levels in aged skeletal muscles while decreasing ROS levels^53^. In the same year, McCully’s group evaluated the therapeutic potential of mitochondrial transplantation in a mouse model of acute limb ischemia (ALI), which leads to loss of muscle viability and function^54^. The injection of mitochondria isolated from donor mouse skeletal muscle into the hindlimb muscles of ALI-induced mice resulted in their distribution within myofibers, a decrease in apoptosis, and an increase in hindlimb function and power, as measured by DigiGait^54^. Recently, Alway et al. showed that in a mouse model of muscle injury induced by BaCl_2_, injecting liver-derived mitochondria through the tail vein resulted in preferential uptake by injured myofibers, which was possibly facilitated by the damaged sarcolemma. This uptake significantly improved muscle regeneration, increased muscle weight and fiber size and restored maximal muscle force^55^. Kim et al. also reported that intramuscular injection of mitochondria obtained from human umbilical cord-derived mesenchymal stem cells (UC-MSCs) significantly increased muscle mass by 1.5-fold and reduced the expression of muscle-specific ubiquitin E3-ligases in a dexamethasone-induced rat model of muscle atrophy^56^. Although research on skeletal muscle-targeted mitochondrial transplantation is still in its early stage, the results thus far suggest that mitochondrial treatment is a promising therapeutic strategy to accelerate the recovery of muscle function and mass.

### Clinical trials

The encouraging results of mitochondrial transplantation in animal models of diseases have led to several human trials. Here, we identified registered clinical trials based on the information provided by ClinicalTrials.gov with search terms for intervention/treatment, including “mitochondria transplantation” or “mitochondria injection”. At the time that this manuscript was revised, another review discussing clinical trials using mitochondrial transplantation has been published^57^. To date, two human trials involving mitochondrial transplantation have been completed, which include the microinjection of mitochondria into oocytes to treat infertility and the infusion of mitochondria-transplanted/augmented hematopoietic stem cells to treat Pearson syndrome (Table 3). More importantly, four trials involving systemic or tissue-specific injection of isolated mitochondria, which align more closely with the focus of this review, are currently recruiting or enrolling by invitation (Table 3). We will introduce these 4 clinical trials in the following paragraphs.

A clinical trial sponsored by Boston Children’s Hospital is recruiting participants to assess the effects of autologous mitochondrial transplantation on myocardial I/R injury and extracorporeal membrane oxygenation (ECMO) complications (NCT02851758). The mitochondria will be isolated from the skeletal muscle of the chest wall and administered to the ischemic myocardium by direct injection (for surgical reoperation subjects) or intracoronary infusion (for catheterization subjects). The outcomes will be determined by safety, improvements in ventricular function, and the ability to be removed from ECMO support.

A clinical trial held at the University of Washington is recruiting subjects to investigate the effects of autologous mitochondrial transplantation on brain ischemia for the first time (NCT04998357). The mitochondria will be isolated from muscle tissues adjacent to the surgical access site and will be infused into an artery in the brain by a microcatheter during standard-of-care endovascular reperfusion therapy. For the outcome measures, investigators will monitor adverse effects associated with mitochondrial infusion and check for a reduction in infarct volume.

A human trial in South Korea sponsored by Paean Biotechnology Inc. is enrolling by invitation to evaluate the safety, tolerability, and efficacy of the transplantation of PN-101 (mitochondria isolated from allogeneic UC-MSCs) in subjects with refractory polymyositis or dermatomyositis (NCT04976140). In mice, PN-101 was shown to reduce pathological inflammatory responses by blocking the nuclear factor kappa B (NFκB) signaling pathway^58^. The mitochondria will be administered intravenously, and the primary outcome will be measured by dose-limiting toxicity (DLT) and International Myositis Assessment and Clinical Studies Group-total improvement score (IMACS-TIS).

Finally, investigators at Tehran University of Medical Sciences are recruiting patients to determine the therapeutic effects of transplanting autologous mitochondria and/or MSC-derived exosomes on myocardial infarction, ischemia, and stunning (NCT05669144). Exosomes-only, mitochondria-only, exosomes plus mitochondria, or placebo will be administered through intracoronary and intramyocardial injection. The primary outcome measures include left ventricle ejection fraction and allergic reactions.

## Conclusion and future perspectives

**Fig. 1:**
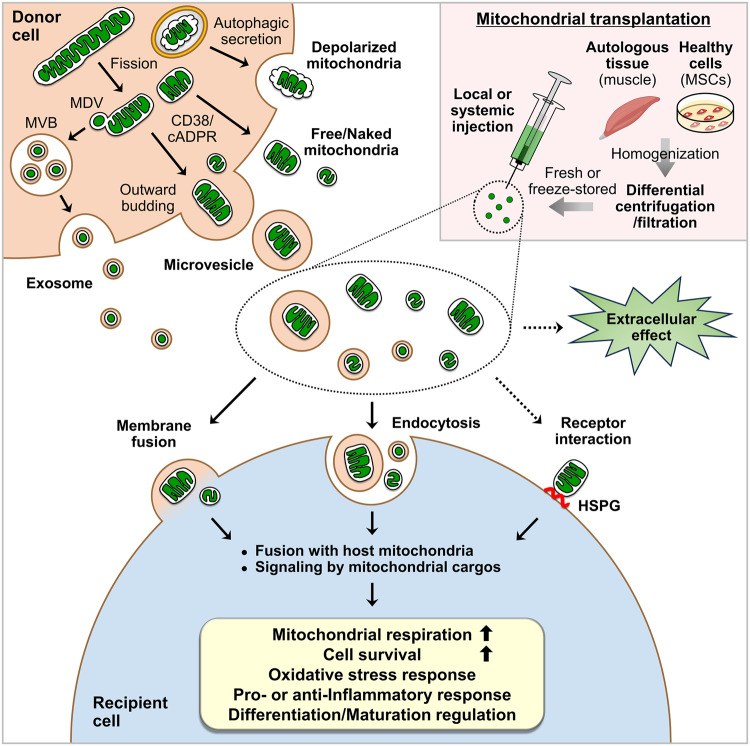
Mitochondrial secretion, transplantation, and biological effects on target cells. Donor cells extracellularly secrete microvesicles containing mitochondria through outward budding, exosomes containing mitochondrial-derived vesicles (MDVs) through the fusion of multivesicular bodies (MVBs) with the plasma membrane, free/naked mitochondria through an unclarified mechanism, or depolarized mitochondria through the secretory autophagy pathway. Mitochondrial fission and CD38/cADPR signaling have been suggested to mediate extracellular mitochondrial secretion. Mitochondrial transplantation involves the isolation of mitochondria from autologous tissues such as skeletal muscle or healthy cells such as mesenchymal stem cells (MSCs) via differential centrifugation or filtration methods and subsequent local or systemic administration. Although the administration of freeze-stored mitochondria has been described, the injection of freshly isolated mitochondria appears to be ideal. Mitochondria that are secreted extracellularly or introduced exogenously can be taken up by recipient cells through membrane fusion or endocytosis. Extracellular mitochondria may also interact with recipient cell surface receptors such as heparan sulfate proteoglycans (HSPGs) for uptake. Once inside the cells, the mitochondria integrate with the host mitochondrial network or activate signaling pathways mediated by their cargo. Although further investigation is needed, secreted or transplanted mitochondria have also been suggested to exert extracellular effects. Overall, these reactions elicit major biological effects on recipient cells, including increases in mitochondrial respiration and cell survival and the regulation of the oxidative stress response, the inflammatory response, and cell differentiation or maturation.

Over the past several years, it has become evident that mitochondria are spontaneously secreted by various cells into the extracellular space and are transferred to recipient cells (Fig. 1). The field of extracellular mitochondrial secretion and transfer has increasingly gained attention partly because (1) a significant advancement in imaging technology has led to a more qualitative way to detect extracellular mitochondrial release by different cell types; (2) the development of various mitochondrial reporter mice, such as PHaM mitoDendra2^59^, C57BL/6J^su9-dsRed237^, adipo-mitoFlag^38^, MitoFat^60^, and *Col1a1-Cre; Igs1*^*CKI-mitoGFP/+*27^ (Table 1), which has allowed greater experimental and technical convenience in the analysis of mitochondrial secretion and transfer in vivo; and 3) the potential therapeutic applications because mitochondria released by donor cells have been shown to enter recipient cells to enhance mitochondrial bioenergetics and cellular functions. Similar to those secreted extracellularly, mitochondria that are transferred exogenously have been shown to regulate mitochondrial metabolism, the inflammatory response, or the differentiation and maturation of recipient cells. This regulation can occur through their integration into the host mitochondrial network, signaling by mitochondrial cargos, or other unspecified extracellular effects (Fig. 1). The promising outcomes of exogenous mitochondrial transfer in animal models of diseases have led to the development of clinical trials with mitochondrial transplantation-based therapeutic interventions. However, research on the mechanisms of the secretion and transfer of mitochondria is still in its early stages, and several critical questions remain to be answered in future investigations.

First, the molecular mechanisms specific to extracellular mitochondrial secretion are largely unknown. While CD38/cADPR signaling has been shown to promote extracellular mitochondrial release in astrocytes and osteoblasts^26,27^, whether it plays the same role in other cells that highly express CD38, such as immune cells, needs further examination. Future research could also focus on identifying additional pathways that commonly regulate mitochondrial secretion by different cell types. Furthermore, mechanisms that differentiate the secretion of free/naked mitochondria from vesicle-enclosed mitochondria also require additional investigation.

Second, it is still unclear whether there are mechanisms that allow extracellularly released mitochondria to target specific cell types. Recently, mitochondria released by adipocytes were shown to preferentially target macrophages among various clusters of cells in adipose tissues^60^, and cellular heparan sulfates were thought to act as receptors to mediate the specific uptake of extracellular mitochondria by macrophages^61^ and human HepG2 cells^62^ (Fig. 1). However, it is also possible that specific surface molecules or proteins are expressed on extracellularly secreted mitochondria to direct their interaction with the recipient cell membrane. Identifying these molecules may allow for the modification or genetic engineering of isolated mitochondria to improve the target specificity of mitochondrial transplantation therapies and minimize off-target effects.

Third, future research should focus on developing techniques to enhance the purity of isolated mitochondria for biological/biochemical analysis and exogenous delivery. While differential centrifugation and differential filtration are the most popular methods used to isolate mitochondria for therapeutic transfer because they are simple and quick processes, they likely also yield nonmitochondrial particles that may contribute to undesired effects. Flow cytometry-based sorting of mitochondria significantly increases purity and allows precise quantification^63^ but takes relatively longer to obtain a sufficient number of mitochondria and requires a specialized device, making it applicable for biochemical analysis of pure mitochondria but suboptimal for clinical situations where fresh mitochondria are expected to be rapidly isolated. The development of strategies to enhance the long-term storage of viable mitochondria for off-the-shelf use would partly overcome this limitation.

Finally, mitochondrial transplantation protocols, including methods, time point, frequency, and dose of administration, needs further establishment and optimization for long-term efficacy. Whether treatment with mitochondria plus other drugs can enhance mitochondrial transfer efficiency or function may also be investigated. The transplantation of cells that are naturally capable of mitochondrial transfer may prolong the effects of mitochondrial delivery and minimize the rejection of exogenous mitochondria. In this regard, in vivo transplantation of CD34+ hematopoietic stem and progenitor cells (HSPCs) augmented with normal exogenous mitochondria ex vivo induced long-term persistence (up to 4.5 months post transplantation) of exogenous mitochondrial transfer from HSPCs to myeloid and B cells in a mouse model of mitochondrial dysfunction^64^. Transplantation of CD34+ stem cells enriched with mitochondria is currently being examined in a clinical trial in pediatric patients with Pearson syndrome (Table 3). Additionally, whether artificially packaging mitochondria in vesicles for transplantation enhances mitochondrial stability or uptake may be tested in the future as mitochondria inside EVs have been suggested to be more resistant to the extracellular environment containing high levels of Ca^2+^ and oxidative stress than free isolated mitochondria^65^.

In conclusion, extracellular mitochondrial secretion, their transfer to recipient cells, and mitochondrial transplantation are increasingly gaining attention for their potential in a variety of therapeutic settings. Understanding the mechanisms and biological effects of the extracellular secretion and transfer of mitochondria, which still require extensive research, serves as a theoretical basis for the development of successful mitochondrial transplantation strategies. Therefore, future efforts should focus on unraveling the molecular and cellular mechanisms of extracellular mitochondrial secretion and transfer, as well as methods to improve the efficiency and efficacy of mitochondrial transplantation therapy.

## References

[1] Monzel AS, Enriquez JA, Picard M (2023). Multifaceted mitochondria: moving mitochondrial science beyond function and dysfunction. Nat Metab.

[2] McArthur, K. et al. BAK/BAX macropores facilitate mitochondrial herniation and mtDNA efflux during apoptosis. *Science* **359**, eaao6047 (2018). 10.1126/science.aao6047 29472455

[3] Baughman JM (2011). Integrative genomics identifies MCU as an essential component of the mitochondrial calcium uniporter. Nature.

[4] Handy DE, Loscalzo J (2012). Redox regulation of mitochondrial function. Antioxid Redox Signal.

[5] Yasukawa K (2009). Mitofusin 2 inhibits mitochondrial antiviral signaling. Sci Signal.

[6] Dohla J (2022). Metabolic determination of cell fate through selective inheritance of mitochondria. Nat Cell Biol.

[7] Csordas G, Weaver D, Hajnoczky G (2018). Endoplasmic Reticulum-Mitochondrial Contactology: Structure and signaling functions. Trends Cell Biol..

[8] Kleele T (2021). Distinct fission signatures predict mitochondrial degradation or biogenesis. Nature.

[9] Giacomello M, Pyakurel A, Glytsou C, Scorrano L (2020). The cell biology of mitochondrial membrane dynamics. Nat Rev Mol Cell Biol.

[10] Soubannier V (2012). A vesicular transport pathway shuttles cargo from mitochondria to lysosomes. Curr Biol.

[11] McLelland GL, Lee SA, McBride HM, Fon EA (2016). Syntaxin-17 delivers PINK1/parkin-dependent mitochondrial vesicles to the endolysosomal system. J Cell Biol.

[12] Mohanty A, Zunino R, Soubannier V, Dilipkumar S (2021). A new functional role of mitochondria-anchored protein ligase in peroxisome morphology in mammalian cells. J Cell Biochem.

[13] Chaiyarit S, Thongboonkerd V (2023). Mitochondria-derived vesicles and their potential roles in kidney stone disease. J Transl Med..

[14] Rosina M (2022). Ejection of damaged mitochondria and their removal by macrophages ensure efficient thermogenesis in brown adipose tissue. Cell Metab..

[15] Hayakawa K (2018). Protective effects of endothelial progenitor cell-derived extracellular mitochondria in brain endothelium. Stem Cells.

[16] Boudreau LH (2014). Platelets release mitochondria serving as substrate for bactericidal group IIA-secreted phospholipase A2 to promote inflammation. Blood.

[17] van Niel G, D’Angelo G, Raposo G (2018). Shedding light on the cell biology of extracellular vesicles. Nat Rev Mol Cell Biol..

[18] Dixson AC, Dawson TR, Di Vizio D, Weaver AM (2023). Context-specific regulation of extracellular vesicle biogenesis and cargo selection. Nat Rev Mol Cell Biol.

[19] Zhang H (2018). Identification of distinct nanoparticles and subsets of extracellular vesicles by asymmetric flow field-flow fractionation. Nat Cell Biol.

[20] Ma L (2015). Discovery of the migrasome, an organelle mediating release of cytoplasmic contents during cell migration. Cell Res.

[21] Zhou X (2023). MitoEVs: A new player in multiple disease pathology and treatment. J Extracell Vesic.

[22] Liu D (2020). The existence and function of mitochondrial component in extracellular vesicles. Mitochondrion.

[23] Liu Z, Sun Y, Qi Z, Cao L, Ding S (2022). Mitochondrial transfer/transplantation: an emerging therapeutic approach for multiple diseases. Cell Biosci..

[24] Spees JL, Olson SD, Whitney MJ, Prockop DJ (2006). Mitochondrial transfer between cells can rescue aerobic respiration. Proc Natl Acad Sci USA.

[25] Phinney DG (2015). Mesenchymal stem cells use extracellular vesicles to outsource mitophagy and shuttle microRNAs. Nat Commun.

[26] Hayakawa K (2016). Transfer of mitochondria from astrocytes to neurons after stroke. Nature.

[27] Suh J (2023). Mitochondrial fragmentation and donut formation enhance mitochondrial secretion to promote osteogenesis. Cell Metab..

[28] Sun L (2002). A novel mechanism for coupling cellular intermediary metabolism to cytosolic Ca2+ signaling via CD38/ADP-ribosyl cyclase, a putative intracellular NAD+ sensor. FASEB J..

[29] Moridera K (2020). Skeletal unloading reduces cluster of differentiation (CD) 38 expression in the bone marrow and osteoblasts of mice. J Orthop Sci..

[30] Sun L (2003). Disordered osteoclast formation and function in a CD38 (ADP-ribosyl cyclase)-deficient mouse establishes an essential role for CD38 in bone resorption. FASEB J..

[31] Nakajima A, Kurihara H, Yagita H, Okumura K, Nakano H (2008). Mitochondrial Extrusion through the cytoplasmic vacuoles during cell death. J Biol Chem..

[32] Tan HWS (2022). A degradative to secretory autophagy switch mediates mitochondria clearance in the absence of the mATG8-conjugation machinery. Nat Commun..

[33] Choong CJ (2021). Alternative mitochondrial quality control mediated by extracellular release. Autophagy.

[34] Nicolas-Avila JA (2020). A Network of Macrophages Supports Mitochondrial Homeostasis in the Heart. Cell.

[35] Zheng D (2021). Mesenchymal stem cell-derived microvesicles improve intestinal barrier function by restoring mitochondrial dynamic balance in sepsis rats. Stem Cell Res Ther..

[36] Peruzzotti-Jametti L (2021). Neural stem cells traffic functional mitochondria via extracellular vesicles. PLoS Biol..

[37] Levoux J (2021). Platelets Facilitate the Wound-Healing Capability of Mesenchymal Stem Cells by Mitochondrial Transfer and Metabolic Reprogramming. Cell Metab..

[38] Crewe C (2021). Extracellular vesicle-based interorgan transport of mitochondria from energetically stressed adipocytes. Cell Metab..

[39] Hough KP (2018). Exosomal transfer of mitochondria from airway myeloid-derived regulatory cells to T cells. Redox Biol..

[40] Morrison TJ (2017). Mesenchymal Stromal Cells Modulate Macrophages in Clinically Relevant Lung Injury Models by Extracellular Vesicle Mitochondrial Transfer. Am J Respir Crit Care Med..

[41] Xia L (2022). AdMSC-derived exosomes alleviate acute lung injury via transferring mitochondrial component to improve homeostasis of alveolar macrophages. Theranostics.

[42] Garcia-Martinez I (2016). Hepatocyte mitochondrial DNA drives nonalcoholic steatohepatitis by activation of TLR9. J Clin Invest..

[43] Maeda A, Fadeel B (2014). Mitochondria released by cells undergoing TNF-alpha-induced necroptosis act as danger signals. Cell Death Dis..

[44] McCully JD (2009). Injection of isolated mitochondria during early reperfusion for cardioprotection. Am J Physiol Heart Circ Physiol..

[45] Masuzawa A (2013). Transplantation of autologously derived mitochondria protects the heart from ischemia-reperfusion injury. Am J Physiol Heart Circ Physiol..

[46] Kaza AK (2017). Myocardial rescue with autologous mitochondrial transplantation in a porcine model of ischemia/reperfusion. J Thorac Cardiovasc Surg.

[47] Doulamis IP (2020). Mitochondrial transplantation for myocardial protection in diabetic hearts. Eur J Cardiothorac Surg.

[48] Huang PJ (2016). Transferring Xenogenic Mitochondria Provides Neural Protection Against Ischemic Stress in Ischemic Rat Brains. Cell Transpl..

[49] Zhang Z (2019). Muscle-derived autologous mitochondrial transplantation: A novel strategy for treating cerebral ischemic injury. Behav Brain Res.

[50] Nakamura Y, Lo EH, Hayakawa K (2020). Placental Mitochondria Therapy for cerebral ischemia-reperfusion injury in mice. Stroke.

[51] Lee EH (2023). Primary astrocytic mitochondrial transplantation ameliorates ischemic stroke. BMB Rep..

[52] Xie Q (2021). Mitochondrial transplantation attenuates Cerebral Ischemia-Reperfusion Injury: Possible involvement of mitochondrial component separation. Oxid Med. Cell Longev..

[53] Zhao Z, Yu Z, Hou Y, Zhang L, Fu A (2020). Improvement of cognitive and motor performance with mitotherapy in aged mice. Int J Biol Sci..

[54] Orfany A (2020). Mitochondrial transplantation ameliorates acute limb ischemia. J Vasc Surg..

[55] Alway SE (2023). Mitochondria transplant therapy improves regeneration and restoration of injured skeletal muscle. J Cachexia Sarcopenia Muscle.

[56] Kim, MJ., Lee, JM, Min, K & Choi, YS. Xenogeneic transplantation of mitochondria induces muscle regeneration in an in vivo rat model of dexamethasone-induced atrophy. *J Muscle Res Cell Motil*10.1007/s10974-023-09643-7. (2023) 10.1007/s10974-023-09643-7 36802005

[57] Kim JS, Lee S, Kim WK, Han BS (2023). Mitochondrial transplantation: an overview of a promising therapeutic approach. BMB Rep..

[58] Yu SH (2022). Human umbilical cord mesenchymal stem cell-derived mitochondria (PN-101) attenuate LPS-induced inflammatory responses by inhibiting NFkappaB signaling pathway. BMB Rep..

[59] Thomas MA (2022). Human mesenchymal stromal cells release functional mitochondria in extracellular vesicles. Front Bioeng Biotechnol..

[60] Borcherding N (2022). Dietary lipids inhibit mitochondria transfer to macrophages to divert adipocyte-derived mitochondria into the blood. Cell Metab..

[61] Brestoff JR (2021). Intercellular Mitochondria transfer to macrophages regulates white adipose tissue homeostasis and is impaired in obesity. Cell Metab..

[62] Kesner EE, Saada-Reich A, Lorberboum-Galski H (2016). Characteristics of mitochondrial transformation into human cells. Sci Rep..

[63] MacDonald JA (2019). A nanoscale, multi-parametric flow cytometry-based platform to study mitochondrial heterogeneity and mitochondrial DNA dynamics. Commun Biol..

[64] Jacoby E (2021). Mitochondrial augmentation of CD34(+) cells from healthy donors and patients with mitochondrial DNA disorders confers functional benefit. NPJ Regen Med.

[65] Ikeda G (2021). Mitochondria-rich extracellular vesicles from autologous stem cell-derived cardiomyocytes restore energetics of ischemic Myocardium. J. Am Coll Cardiol..

[66] Jackson MV (2016). Mitochondrial transfer via tunneling nanotubes is an important mechanism by which mesenchymal stem cells enhance macrophage phagocytosis in the in vitro and in vivo models of ARDS. Stem Cells.

[67] Ko JH, Kim HJ, Jeong HJ, Lee HJ, Oh JY (2020). Mesenchymal stem and stromal cells harness macrophage-derived amphiregulin to maintain tissue homeostasis. Cell Rep..

[68] Wang Y (2020). Activation of astrocytic sigma-1 receptor exerts antidepressant-like effect via facilitating CD38-driven mitochondria transfer. Glia.

[69] D’Acunzo, P et al. Mitovesicles are a novel population of extracellular vesicles of mitochondrial origin altered in Down syndrome. *Sci Adv* **7**, eabe5085 (2021). 10.1126/sciadv.abe5085 PMC7880603 33579698

[70] Unuma K, Aki T, Funakoshi T, Hashimoto K, Uemura K (2015). Extrusion of mitochondrial contents from lipopolysaccharide-stimulated cells: Involvement of autophagy. Autophagy.

[71] Cai, Y et al. Mitochondrial DNA-enriched microparticles promote acute-on-chronic alcoholic neutrophilia and hepatotoxicity. *JCI Insight* **2**, e92634 (2017). 10.1172/jci.insight.92634 PMC5518559 28724791

[72] Leermakers PA (2020). Iron deficiency-induced loss of skeletal muscle mitochondrial proteins and respiratory capacity; the role of mitophagy and secretion of mitochondria-containing vesicles. FASEB J..

[73] Puhm F (2019). Mitochondria are a subset of extracellular vesicles released by activated monocytes and induce Type I IFN and TNF responses in endothelial cells. Circ Res..

[74] Abad E, Lyakhovich A (2022). Movement of Mitochondria with mutant DNA through extracellular vesicles helps cancer cells acquire Chemoresistance. ChemMedChem.

[75] Takenaga K, Koshikawa N, Nagase H (2021). Intercellular transfer of mitochondrial DNA carrying metastasis-enhancing pathogenic mutations from high- to low-metastatic tumor cells and stromal cells via extracellular vesicles. BMC Mol Cell Biol..

[76] Cowan DB (2016). Intracoronary delivery of mitochondria to the ischemic heart for cardioprotection. PLoS One.

[77] Moskowitzova K (2019). Mitochondrial transplantation prolongs cold ischemia time in murine heart transplantation. J Heart Lung Transpl..

[78] Guariento A (2020). Preischemic autologous mitochondrial transplantation by intracoronary injection for myocardial protection. J Thorac Cardiovasc Surg..

[79] Weixler V (2021). Autogenous mitochondria transplantation for treatment of right heart failure. J Thorac Cardiovasc Surg..

[80] Alemany, VS et al. Mitochondrial transplantation preserves myocardial function and viability in pediatric and neonatal pig hearts donated after circulatory death. *J Thorac Cardiovasc Surg.* **167**, e6–e21 (2023). 10.1016/j.jtcvs.2023.05.010 37211245

[81] Mokhtari B, Hamidi M, Badalzadeh R, Mahmoodpoor A (2023). Mitochondrial transplantation protects against sepsis-induced myocardial dysfunction by modulating mitochondrial biogenesis and fission/fusion and inflammatory response. Mol Biol Rep.

[82] Babenko, VA et al. Miro1 enhances mitochondria transfer from multipotent Mesenchymal Stem Cells (MMSC) to neural cells and improves the efficacy of cell recovery. *Molecules* **23**, 687 (2018). 10.3390/molecules23030687 PMC6017474 29562677

[83] Pourmohammadi-Bejarpasi Z (2020). Mesenchymal stem cells-derived mitochondria transplantation mitigates I/R-induced injury, abolishes I/R-induced apoptosis, and restores motor function in acute ischemia stroke rat model. Brain Res Bull..

[84] Bamshad C (2023). Human umbilical cord-derived mesenchymal stem cells-harvested mitochondrial transplantation improved motor function in TBI models through rescuing neuronal cells from apoptosis and alleviating astrogliosis and microglia activation. Int Immunopharmacol..

[85] Chang JC (2016). Allogeneic/xenogeneic transplantation of peptide-labeled mitochondria in Parkinson’s disease: restoration of mitochondria functions and attenuation of 6-hydroxydopamine-induced neurotoxicity. Transl Res..

[86] Chang JC (2021). Intranasal delivery of mitochondria for treatment of Parkinson’s Disease model rats lesioned with 6-hydroxydopamine. Sci Rep.

[87] Nitzan K (2019). Mitochondrial transfer ameliorates cognitive deficits, neuronal loss, and gliosis in Alzheimer’s disease mice. J Alzheimers Dis..

[88] Wang Y (2019). Mitochondrial transplantation attenuates lipopolysaccharide- induced depression-like behaviors. Prog Neuropsychopharmacol Biol Psychiatry.

[89] Zhang Z (2022). Hippocampal mitochondrial transplantation alleviates age-associated cognitive decline via enhancing Wnt signaling and neurogenesis. Comput Intell Neurosci..

[90] Gollihue JL (2018). Effects of mitochondrial transplantation on bioenergetics, cellular incorporation, and functional recovery after spinal cord injury. J Neurotrauma.

[91] Li H (2019). Mitochondrial transfer from bone marrow mesenchymal stem cells to motor neurons in spinal cord injury rats via gap junction. Theranostics.

[92] Lin MW (2022). Mitochondrial transplantation attenuates neural damage and improves locomotor function after traumatic spinal cord injury in rats. Front Neurosci.

[93] Lin HC, Liu SY, Lai HS, Lai IR (2013). Isolated mitochondria infusion mitigates ischemia-reperfusion injury of the liver in rats. Shock.

[94] Fu A, Shi X, Zhang H, Fu B (2017). Mitotherapy for fatty liver by intravenous administration of exogenous mitochondria in male mice. Front Pharmacol..

[95] Shi X (2018). Treatment of acetaminophen-induced liver injury with exogenous mitochondria in mice. Transl Res..

[96] Ulger O (2021). The effects of mitochondrial transplantation in acetaminophen-induced liver toxicity in rats. Life Sci..

[97] Lu T (2022). Extracellular vesicles derived from mesenchymal stromal cells as nanotherapeutics for liver ischaemia-reperfusion injury by transferring mitochondria to modulate the formation of neutrophil extracellular traps. Biomaterials.

[98] Islam MN (2012). Mitochondrial transfer from bone-marrow-derived stromal cells to pulmonary alveoli protects against acute lung injury. Nat Med..

[99] Zhang F (2023). TFAM-Mediated mitochondrial transfer of MSCs improved the permeability barrier in sepsis-associated acute lung injury. Apoptosis.

[100] Moskowitzova K (2020). Mitochondrial transplantation enhances murine lung viability and recovery after ischemia-reperfusion injury. Am J Physiol Lung Cell Mol Physiol..

[101] Huang T (2021). Iron oxide nanoparticles augment the intercellular mitochondrial transfer-mediated therapy. Sci Adv..

[102] Zou X (2018). Renal scattered tubular-like cells confer protective effects in the stenotic murine kidney mediated by release of extracellular vesicles. Sci Rep..

[103] Konari N, Nagaishi K, Kikuchi S, Fujimiya M (2019). Mitochondria transfer from mesenchymal stem cells structurally and functionally repairs renal proximal tubular epithelial cells in diabetic nephropathy in vivo. Sci Rep..

[104] Jabbari H (2020). Mitochondrial transplantation ameliorates ischemia/reperfusion-induced kidney injury in rat. Biochim Biophys. Acta Mol. Basis Dis..

[105] Doulamis IP (2020). Mitochondrial transplantation by intra-arterial injection for acute kidney injury. Am J Physiol Ren Physiol..

[106] Zhao M (2021). Mesenchymal stem cell-derived extracellular vesicles attenuate mitochondrial damage and inflammation by stabilizing mitochondrial DNA. ACS Nano.

[107] Yuan Y (2021). Mitochondrial transfer from mesenchymal stem cells to macrophages restricts inflammation and alleviates kidney injury in diabetic nephropathy mice via PGC-1alpha activation. Stem Cells.

[108] Sun J (2022). High-efficiency quantitative control of mitochondrial transfer based on droplet microfluidics and its application on muscle regeneration. Sci Adv.

[109] Guo Y (2020). Mitochondria transfer enhances proliferation, migration, and osteogenic differentiation of bone marrow mesenchymal stem cell and promotes bone defect healing. Stem Cell Res Ther..

[110] Lee AR (2022). Mitochondrial transplantation ameliorates the development and progression of Osteoarthritis. Immune Netw..

